# Unveiling the Mysteries of RAS Signaling: A Journey of Discovery and Breakthroughs

**DOI:** 10.3390/genes14111987

**Published:** 2023-10-25

**Authors:** Esther Castellano

**Affiliations:** Tumour-Stroma Signalling Laboratory, Centro de Investigación del Cáncer, Instituto de Biología Molecular y Celular del Cáncer, Consejo Superior de Investigaciones Científicas (CSIC)—Universidad de Salamanca, Campus Miguel de Unamuno, 37007 Salamanca, Spain; ecastellano@usal.es

In the realm of molecular biology, few terms evoke as much curiosity, fascination, and determination as RAS. For more than four decades, researchers have relentlessly pursued a deeper understanding of RAS signaling, unravelling its intricate web of interactions in health and disease. Today, we stand at a remarkable juncture, where ground-breaking advancements have allowed us to finally drug this once elusive oncogene. With this Special Issue on RAS signaling in health and disease, we celebrate the culmination of efforts that have brought us to this historic milestone.

One of the key aspects of RAS signaling lies in its multimeric interactions on the cellular membrane. The review article “Ras Multimers on the Membrane: Many Ways for a Heart-to-Heart Conversation” [1] sheds light on the intricate dynamics of these interactions, elucidating how they contribute to cellular communication and physiological processes.

The emergence of RAS dimers as pivotal players in the RAS-ERK pathway has opened up new avenues for exploration. “RAS Dimers: The Novice Couple at the RAS-ERK Pathway Ball” [2] explores the significance of these interactions and their impact on signal transduction, shedding light on potential therapeutic interventions.

PI3K has emerged as a critical component in the RAS signaling network, influencing diverse cellular processes. “The Importance of Being PI3K in the RAS Signaling Network” [3] delves into the intricate crosstalk between PI3K and RAS, uncovering novel insights into the regulation of RAS-mediated cellular responses.

After years of intense research and ingenuity, we have finally witnessed ground-breaking advances in the targeting of RAS in cancer. “Drugging the Undruggable: Advances on RAS Targeting in Cancer” [4] showcases these transformative breakthroughs, highlighting the novel strategies and therapeutic modalities that have opened new frontiers in cancer treatment.

The convergence of the RAS and RHO signaling pathways presents a captivating nexus with profound implications for cellular transformation, motility, and contraction. “The Crossroads between RAS and RHO Signaling Pathways in Cellular Transformation, Motility, and Contraction” [5] delves into the intricate interplay between these pathways, providing invaluable insights into the mechanisms underlying cellular dynamics.

Ras GTPases have emerged as critical contributors to kidney fibrosis, a condition of significant clinical importance. “Dissecting the Involvement of Ras GTPases in Kidney Fibrosis” [6] disentangles the intricate roles of Ras GTPases in this complex pathology, offering potential avenues for therapeutic intervention.

As we reflect on the journey of RAS research, “40 Years of RAS—A Historic Overview” [7] offers a panoramic view of the significant milestones, the challenges faced, and the remarkable progress made in understanding RAS signaling. It pays homage to the pioneers in this field and acknowledges the collective efforts that have brought us to this remarkable moment in RAS research.

While oncogenic RAS has been the focus of intense scrutiny, the role of wild-type RAS in the transformation process cannot be overlooked. “The Role of Wild-Type RAS in Oncogenic RAS Transformation” [8] sheds light on the intricate interplay between wild-type and oncogenic RAS, enriching our comprehension of RAS-mediated oncogenesis.

The RAF signaling pathways hold immense potential as therapeutic targets for blocking oncogenic RAS signaling. “Hidden Targets in RAF Signaling Pathways to Block Oncogenic RAS Signaling” [9] uncovers the lesser-known aspects of RAF signaling, identifying promising avenues for therapeutic intervention.

With these extraordinary review articles on RAS signaling in health and disease, we celebrate the tireless efforts of scientists, researchers, and clinicians who have dedicated their careers to unlocking the mysteries of RAS. These are special times indeed, as we are witnessing long-awaited breakthroughs in drugging the “undruggable” oncogene. Let us seize this moment of triumph to not only reflect on the arduous journey but also to envision the future possibilities that lie ahead, as we continue to decode the complexities of RAS signaling and strive to transform lives through scientific discoveries.

Together, let us embark on this fascinating exploration of RAS signaling, uniting our knowledge and embracing the special times we are living in.
